# Cost-effectiveness Analysis of Denosumab in the Prevention of Skeletal-related Events in Patients with Prostate Cancer in Kazakhstan

**DOI:** 10.5195/cajgh.2014.154

**Published:** 2014-12-12

**Authors:** Carina Bektur, Talgat Nurgozhin

**Affiliations:** Center for Life Sciences, Nazarbayev University, Astana, Kazakhstan

**Keywords:** prostate cancer, bone mass loss, denosumab, zoledronic, cost-effectiveness

## Abstract

**Introduction:**

Bone mass loss (BML) is one of the adverse effects of oncological chemotherapy, especially in cases of hormonal types of cancer, such as a prostate cancer (PC). BML is strongly associated with skeletal-related events (SREs), therefore decreasing the quality of patient’s life. Denosumab shows an advantage over zoledronic acid (ZA) in delaying the first onset of SREs and subsequent SREs in adults with PC in several phase III clinical trials. Since generic ZA recently became available, the purpose of the present study was to assess the cost-effectiveness of denosumab vs. brand or generic ZA in the prevention of SREs in Kazakhstani patients with PC.

**Methods:**

A Markov model was constructed in Tree-Age Pro 2013 software program with 4-week model cycles to analyze the cost-effectiveness of the treatments from the perspective of Ministry of Health (MoH) over a 10-year PC cohort. Direct costs (in Kazakhstani monetary units “tenge” in 2014) included costs of drug, SRE (pathologic fracture, surgery to bone, radiation to bone, spinal cord compression), and adverse events treatment. All costs were discounted for 3% per year. Effectiveness was appraised based on the number of SREs. Health states were defined according to SRE occurrence, SRE history, and death. The model assumed that a maximum of 1 SRE could occur in each cycle. Transition probabilities were derived from the relevant phase III trials. Results were present in the incremental total cost per SRE avoided. One-way sensitivity analyses were performed to examine the robustness of the model.

**Results:**

Over the 10-year period, denosumab incurred 103,091 tenge higher costs than brand ZA, 677,133 tenge higher costs than generic ZA, and 0.58 fewer SREs per patient with PC. The estimated incremental total direct costs per SRE avoided with the use of denosumab were 177,743 tenge (instead of brand ZA) and 1,167,470 tenge (instead of generic ZA). Results were robust to one-way sensitivity analyses.

**Conclusions:**

With the assumption that brand and generic ZAs are equally effective in the prevention of SREs in PC patients, denosumab seems to be a cost-effective alternative for brand ZA (insignificant difference in costs – less than 5%) and a costly alternative for generic ZA from the perspective of MoH of Kazakhstan.

